# Sexually transmitted infections among pre-exposure prophylaxis users in a Swedish multi-centre cohort

**DOI:** 10.1093/eurpub/ckaf034

**Published:** 2025-04-04

**Authors:** Tobias Herder, Fredrik Månsson, Petra Tunbäck, Karin Sanner, Magnus Gisslén, Ester Fridenström, Minna Dawar, Susanne Strömdahl

**Affiliations:** Social Medicine & Global Health, Department of Clinical Sciences, Faculty of Medicine, Lund University, Malmö, Sweden; Clinical Infection Medicine, Department of Translational Medicine, Faculty of Medicine, Lund University, Malmö, Sweden; Department of Infectious Diseases, Skåne University Hospital, Malmö, Sweden; Department of Dermatology and Venereology, Institute of Clinical Sciences, Sahlgrenska Academy, University of Gothenburg, Gothenburg, Sweden; Department of Dermatology and Venereology, Sahlgrenska University Hospital, Region Västra Götaland, Gothenburg, Sweden; Department of Dermatology and Venereology, Uppsala University Hospital, Region Uppsala, Sweden; Department of Infectious Diseases, Institute of Biomedicine, Sahlgrenska Academy at University of Gothenburg, Gothenburg, Sweden; Department of Infectious Diseases, Sahlgrenska University Hospital, Region Västra Götaland, Gothenburg, Sweden; Infection Medicine, Department of Medical Sciences, Uppsala University, Uppsala, Sweden; Clinical Infection Medicine, Department of Translational Medicine, Faculty of Medicine, Lund University, Malmö, Sweden; Department of Infectious Diseases, Skåne University Hospital, Malmö, Sweden; Infection Medicine, Department of Medical Sciences, Uppsala University, Uppsala, Sweden; Department of Infectious Diseases, Uppsala University Hospital, Region Uppsala, Sweden; Global and Sexual Health, Department of Global Public Health, Karolinska Institutet, Stockholm, Sweden

## Abstract

An additional upsurge in bacterial STIs has been observed in Sweden following HIV pre-exposure prophylaxis (PrEP) implementation. From a prevention perspective, it is of relevance to optimize testing strategies within PrEP programmes to identify those most at risk. An open retrospective longitudinal observational cohort study was performed at three STI clinics in Uppsala, Gothenburg, and Malmö. A questionnaire and journal data regarding STI were collected from a sample of 199 MSM enrolled in the PrEP programmes and providing informed consent. Incident bacterial STIs during follow-up were analyzed with descriptive statistics, Poisson regression, and Cox regression. Median follow-up time was 632 days. A total of 270 gonorrhoea or chlamydia infections were recorded during PrEP follow-up, compared to 194 cases in the 2-year period prior to enrolment, giving an incidence rate ratio (IRR) of 1.56 (CI 95% 1.30–1.89). The testing frequency increased by 75% (IRR 1.69, CI 95% 1.60–1.90). For diagnoses of active syphilis, the increase was 108% (IRR 2.08. CI 95% 1.04–4.06), compared with a 5-year period preceding enrolment. The hazard ratio of time (days) until a first STI after PrEP initiation was 2.87 (CI 95% 1.72–4.80) for those having had a STI prior to PrEP initiation and 2.06 (CI 95% 1.03–4.13) for those reporting experience of group sex in the past year compared with those who had not. STI prior to PrEP initiation and group sex were associated with STI after initiation of PrEP, factors that could be considered if needing prioritizing the frequency of STI screening.

## Introduction

Oral pre-exposure prophylaxis (PrEP) against HIV is highly effective in preventing HIV acquisition in men who have sex with men (MSM) [1–3]. WHO has recommended PrEP for MSM as part of a comprehensive HIV prevention package since 2014 [4]. Sweden started recommending PrEP in 2017 for individuals at increased risk of contracting HIV [5], the implementation started in 2018 and since then the country has seen a gradual rollout of PrEP prescriptions. However, the accessibility of PrEP in Sweden has encountered variations over time and across different regions. The most recent report from The Swedish Federation for Lesbian, Gay, Bisexual, Transgender, Queer and Intersex Rights (RFSL) estimated that in March 2024, approximately 4944 individuals had received PrEP prescriptions since implementation started, with approximately 3890 current active users [6].

With the introduction of PrEP, the sexual health approaches for MSM have shifted. Previously, condom use was a central preventive tool for HIV that also had an effect on bacterial STIs, while bacterial STIs could now be seen as separated from HIV in the preventive packages. There has been expressed concerns for risk compensation in the form of decreased condom use among PrEP users, but there is no conclusive evidence of risk compensation regarding changes in condom use or the number of sexual partners [7, 8]. Taking a more holistic approach to sexual health, one can argue that PrEP contributes to overall sexual health, as a result of reduced HIV-related anxiety and fear [9, 10].

Bacterial STIs have nevertheless become an increasing concern for the MSM group, with trends of increasing incidence rates observed in Sweden and other countries of Europe [11] trends that notably began before the introduction of PrEP [12]. With finite resources for STI testing and care, PrEP implementation has placed extra pressure on healthcare services in some clinics [13]. Current Swedish clinical recommendations advise PrEP users to undergo screening for chlamydia and gonorrhoea (in three anatomical sites: urethra, pharynx, rectum) and syphilis every three months [14], resulting in STI screenings for PrEP users consuming a considerable proportion of available clinic resources. While a majority enrolled in the PrEP programmes can be considered to be at substantial risk of bacterial STIs, identifying both those most at risk and those with lower risk to enable individually adapted STI screening frequencies could be of clinical relevance. Individually adapted STI screening frequencies might also be helpful to PrEP users as part of tailoring their care. This is to our knowledge the first study to present data from a cohort of Swedish PrEP users.

The overall aim of the current study was to examine if bacterial STI infection prior to PrEP initiation is a predictor of bacterial STI infection after PrEP initiation in a Swedish cohort of PrEP users at three clinics in Uppsala, Malmö, and Gothenburg. An improved understanding of STI risk among PrEP users could inform future prioritizations in PrEP programmes for MSM, which in turn could enable resources for PrEP scale-up.

## Methods

### Study setting and population

The current study was a multi-centre study at three STI clinics prescribing PrEP in Sweden, one clinic in each of the cities of Uppsala, Gothenburg, and Malmö, coordinated by Uppsala University Hospital. While the clinics in Gothenburg and Malmö are STI clinics with a substantiate number of MSM attendants, all three clinics cater to a wider patient group including non-MSM. The study was an open retrospective longitudinal observational cohort study. Participant inclusion took place between November 2018 and March 2023.

The study included adult individuals (≥18 years) who attended an initial PrEP consultation at one of the participating clinics, had the intention to start PrEP, were HIV seronegative, and who provided written informed consent to participate in the study. Seronegative status at inclusion was assessed by Abbott’s Alinity HIV Ag/Ab Combo assay (Uppsala, Gothenburg) and HIV Combo Vitros 3600 (Malmö).

In the Swedish setting, PrEP is not provided free of charge but is included in the state-sponsored subsidy system which subsidizes medicines above a certain yearly cost which in 2023 was 2600 SEK [15]. STI testing is free, and that has not changed before/after PrEP introduction. Prescription is performed for 3 months at the time and new HIV/STI testing (blood + urine/urethra/pharyngeal/rectal swabs for MSM) is required for a renewed prescription. The testing methods did not differ before/after PrEP initiation and all tests were performed at the three STI clinics as part of clinical practice.

A self-administered questionnaire was collected at participant inclusion. A Journal data review was performed regarding STI testing from continuous follow-up screenings as part of clinical care during enrolment in the PrEP programmes and for 2 years prior for chlamydia and gonorrhoea and 5 years prior for syphilis. Sweden’s initial PrEP guidelines from 2017 specified that a history of STI during these time periods was considered risk factor for HIV, which informed the study design [5]. The questionnaire dataset and journal dataset were anonymized and later combined by using a study-specific anonymous participant number.

Study inclusion and follow-up occurred during the Covid-19 pandemic in Sweden. Inclusion was halted temporarily at all sites during 2020 but already included participants were continuously followed.

The study was conducted in accordance with the Declaration of Helsinki, and ethical approval was obtained from the Regional Ethical Review Board in Uppsala, Sweden (dnr 2018/284 and 2019/00555).

### Measurements

The main outcome studied was defined as the diagnosis of a bacterial STI during follow-up. Chlamydia and gonorrhoea diagnosis are defined as a positive PCR test (pharyngeal or rectal swab, or urine/urethra sample). An automated treponemal test (CLIA Liaison^®^, DiaSorin) was used for initial syphilis screening. Then further testing was performed with TPHA (threshold titre 1/80) for confirmation of the diagnoses and a non-treponemal test (VDRL, threshold titre 1/1) in order to guide patient management decisions [16, 17].

Participants contributed with follow-up time from the date of the first PrEP prescription until either the first bacterial STI infection, the last clinical visit for follow-up, or the date noted in the patient journal for PrEP discontinuation. Reasons for discontinuation could be not wanting to continue to use PrEP or due to geographically moving and changing clinics.

The main explanatory variable, STI diagnosis prior to PrEP initiation, was assessed for 2 years prior to PrEP initiation for gonorrhoea or chlamydia diagnosis, and 5 years prior for syphilis diagnosis, in line with the Swedish PrEP eligibility criteria in the national clinical guidelines at the time of study initiation [5].

Other covariables included in the study include data from a self-administered questionnaire given to the participants at the time of enrolment. Variables concerning behaviours such as number of partners, condom use, drug use, group sex and sex abroad were all self-reported and assessed for the preceding 12 months. Continuous variables such as age and number of partners were dichotomized with the median as the cutting point for the analysis.

### Statistical analysis

Frequency tables of main explanatory variables and covariates were produced to compare the groups based on the main outcome variable, bacterial STI during follow-up. Kaplan-Meyer curves were presented to examine the cumulative incidences of bacterial STIs during follow-up, stratified by STI diagnoses prior to PrEP initiation. Cox proportional hazard regression was conducted to examine hazard ratios for the time until first bacterial STIs during follow-up. Follow-up started on the date of the first PrEP prescription. Participants were censored at either the date of their last follow-up visit to the clinic or the date of PrEP discontinuation. Failure was defined as occurring at the date of the first bacterial STI infection. Proportionalities of hazards were assessed graphically and by using Schoenfeld residuals.

An additional descriptive analysis was performed to examine new episodes of bacterial STIs during follow-up. All statistical analyses were performed using STATA/SE16.1.

## Results

As the current clinical guidelines of PrEP in Sweden focus on MSM specifically, the 199 included participants could be considered belonging to the MSM group, but with a variety of identities and experiences represented including non-binary identifying individuals (*n* = 8) and male-identifying individuals who identified as trans (*n* = 3). All reported sex with men in the past 12 months. A vast majority, 81.8% (*n* = 157), of the participants identified as homosexual, 12.5% (*n* = 24) identified as bisexual, and the reminding 11 individuals (5.7%) who specified a sexual orientation included individuals who stated that they did not define their sexual orientation, queer identity, pansexual, and heterosexual. Regarding sexual positioning at anal sex, a versatile practice as both top (insertive partner) and bottom (receptive partner) was the most common (68.6%) with fewer participants reporting exclusive positioning as top (18.3%) or bottom (13.1%). The median age among participants was 35 years, with the youngest being 21 years and the oldest 70 years. Overall, the educational level was high in the cohort with 68.4% having a university education, and a majority 67.0% were born in Sweden. Sociodemographic characteristics are further detailed in Table 1.

**Table 1.** Frequency table of sociodemographic characteristics, total and stratified by STI diagnosis during follow-up (*N* = 199)

	Total		No STI diagnosis		STI diagnosis	
Total (*N*, %)	199	100.0%	122	61.3%	77	38.7%
Follow-up days (median, IQR)	232	84; 581	241	69; 649	218	100; 463
Total follow-up time (person-years at risk)	209		138		71	
Site of enrolment (*n*, %)						
Uppsala	96	48.4%	73	76.0%	23	24.0%
Malmö	57	28.6%	28	49.1%	29	50.9%
Gothenburg	46	23.1%	21	45.7%	25	54.4%
*Missing*	*0*		*0*		*0*	
Age (median, IQR)	35	30; 45	33.5	28; 43	38	33; 46
Country of birth (*n*, %)						
Outside Sweden	64	33.0%	40	62.5%	24	37.5%
Sweden	130	67.0%	81	62.3%	49	37.7%
*Missing*	*5*		*1*		*4*	
Level of education (*n*, %)						
Up to secondary or vocational	61	31.6%	38	62.3%	23	37.7%
University education	132	68.4%	82	62.1%	50	37.9%
*Missing*	*6*		*2*		*4*	

The median follow-up time for participants after PrEP initiation was 632 days with a total follow-up time for all participants of 129 439 days (354.4 years). The median follow-up time until first reported STI was 232 days, and total follow-up time until first STI was 76 210 days. For the 2-year period prior to PrEP initiation of each participant, a total of 194 chlamydia or gonorrhoea infections were registered, which is an incidence of 48.7 infections per 100 person-years. During follow-up, a total of 270 infections were diagnosed, giving an incidence of 76.3 infections per 100 person years in the sample, an increase of 56.5% (IRR 1.56: CI 95% 1.30–1.89). The test positivity rate for gonorrhoea and chlamydia 2-year prior to PrEP initiation was 23.6% and 21.2% during the PrEP follow-up period (*P* = 0.29).

For the 2-year period prior to PrEP, the 194 chlamydia and gonorrhoea infections were among 84 of the participants, and 191 (96%) of the participants had at least 1 test recorded, with a median of four tests (IQR: 2; 5). During follow-up, the 270 infections were among 71 participants, and 171 of the participants had at least 1 test recorded. Thereby 28 participants (14%) had not yet been tested for chlamydia or gonorrhoea during PrEP follow-up, after baseline testing due to recently initiating PrEP. Multiple episodes of infections were common, with 1–18 separate infections recorded among those participants diagnosed.

There were 820 registered separate test occasions for chlamydia or gonorrhoea 2 year prior to PrEP initiation (ranging from 0 to 20 occasions for a single participant), in the sample of 199 MSM, this gives a test rate of 206 test occasions per 100 person-years. During the total follow-up time after PrEP initiation of the cohort, there were 1275 separate testing occasions registered (ranging from 0 to 32 occasions for one participant). Given a sample of 191 individuals with follow-up tests and a total follow-up time from PrEP initiation until the end of follow-up of 129 439 days (354.4 years), this resulted in an incidence of 360 test occasions per 100 person-years, an increase of 75% (IRR 1.69, CI 95% 1.60–1.91).

For syphilis, 23 active syphilis infections were recorded for the 5-year period prior to PrEP initiation, giving an incidence of 2.3 infections per 100 person-years. During follow-up, 17 active syphilis infections were recorded giving an incidence of 4.8 infections per 100 person-years, an increase of 108% (IRR 2.08 (CI 95% 1.04–4.06).

No participant in the study cohort seroconverted to HIV positivity during follow-up. As shown in Table 2, the proportion of participants with an STI diagnosis during follow-up was higher among those who had an STI diagnosis registered prior to PrEP initiation (51.6%) than among those who did not have an STI prior (26.9%). The median number of partners was also considerably higher among those with STI diagnosis during follow-up (28, IQR 12.5; 51.5) compared to those without STI during follow-up (16, IQR 8; 32). Reporting never or seldom using condoms was more common with regular partners (51.7% as top, 53.0% as bottom) than temporary partners (30.3% as top, 33.1% as bottom), but for three out of the four condom use variables analyzed the proportion with STI during follow-up was higher among those who reported using condoms more often.

**Table 2.** Frequency table of behaviours, preventive measures and STI

	Total		No STI diagnosis		STI diagnosis	
Total	199	100%	122	61.3%	77	38.7%
STI diagnosis prior to PrEP initiation (*n*, %)						
No	104	52.3%	76	73.1%	28	26.9%
Yes	95	47.7%	46	48.4%	49	51.6%
GC/CT diagnosis prior to PrEP initiation (2 years) (*n*, %)						
No	115	57.8%	81	70.4%	34	29.6%
Yes	84	42.2%	41	48.8%	43	51.2%
Syphilis diagnosis prior to PrEP initiation (5 years) (*n*, %)						
No	178	89.5%	115	64.6%	63	35.4%
Yes	21	10.6%	7	33.3%	14	66.7%
Number of male partners, total, 12 months (median, IQR)	17.5	9; 38	14	8; 32	28	12.5; 51.5
Seldom/never use condoms with regular partners as top (*n*, %)^a^						
No—use condoms more often	72	48.3%	39	54.2%	33	45.8%
Yes—seldom/never	77	51.7%	47	61.0%	30	39.0%
Seldom/never use condoms with regular partners as the bottom (*n*, %)^a^						
No—use condoms more often	70	47.0%	37	52.9%	33	47.1%
Yes—seldom/never	79	53.0%	50	63.3%	29	36.7%
Seldom/never use condoms with temporary partners as top (*n*, %)^a^						
No—use condoms more often	108	69.7%	66	61.1%	42	38.9%
Yes—seldom/never	47	30.3%	25	53.2%	22	46.8%
Seldom/never use condoms with temporary partners as bottom (*n*, %)^a^						
No—use condoms more often	99	66.9%	59	59.6%	40	40.4%
Yes—seldom/never	49	33.1%	33	67.4%	16	32.7%
Group sex, 12 months (*n*, %)						
No	64	32.2%	49	76.6%	15	23.4%
Yes	128	64.3%	69	53.9%	59	46.1%
Used poppers during sex, 12 months (*n*, %)						
No	75	38.9%	57	76.0%	18	24.0%
Yes	118	61.1%	63	53.4%	55	46.6%
Used erectile dysfunction drugs during sex, 12 months (*n*, %)						
No	141	73.1%	95	67.4%	46	32.6%
Yes	52	26.9%	25	48.1%	27	51.9%
Used cannabis during sex, 12 months (*n*, %)						
No	160	82.9%	101	63.1%	59	36.9%
Yes	33	17.1%	19	57.6%	14	42.4%
Used non-injecting drugs during sex, 12 months (*n*, %)^b^						
No	169	87.6%	105	62.1%	64	37.9%
Yes	24	12.4%	15	62.5%	9	37.5%
Used injecting drugs during sex, 12 months (*n*, %)^c^						
No	192	99.5%	119	62.0%	73	38.0%
Yes	1	0.5%	1	100.0%	0	0.0%
Sex abroad, 12 months (*n*, %)						
No	66	34.0%	47	71.2%	19	28.8%
Yes	128	66.0%	73	57.0%	55	43.0%
Most recent HIV test, prior to enrolment (*n*, %)						
>6 months before enrolment	30	15.4%	24	80.0%	6	20.0%
≤6 months before enrolment	165	84.6%	97	58.8%	68	41.2%
Self-reported PEP use prior to enrolment (*n*, %)						
No	174	91.6%	108	62.1%	66	37.9%
Yes	16	8.42%	8	50.0%	8	50.0%
Self-reported PrEP use prior to enrolment (*n*, %)						
No	147	85.5%	93	63.3%	54	36.7%
Yes	25	14.5%	16	64.0%	9	36.0%

a For condom use co-variates, the denominator is only participants who reported receptive or insertive anal sex with temporary or regular partners, those not reporting relevant sexual practice are coded and reported as *expected missing*. Total and stratified by STI diagnosis during follow-up (*N* = 199).

b Non-injected cocaine, LSD, GHB, crystal meth, ketamine, crack, amphetamine, and/or ecstasy.

c Injected amphetamine, heroin, crack, and/or crystal meth.

While injecting drug use during sex was uncommon among the participants, other forms of intake of intoxicants or drugs during sex were more common with 61.1% of the participants reporting poppers use, 26.9% the use of erectile dysfunctions drugs such as Viagra, and 12.4% reporting non-injecting heavy drug use including cocaine, GHB ecstasy, LSD, crystal meth, ketamine, crack, and amphetamine. A majority (66%) of the participants reported sex abroad, and group sex was commonly occurring (64.3%) with a larger proportion of STI during follow-up among those who reported group sex (46.6%) than among those who did not report group sex (23.4%).

**Figure 1.**
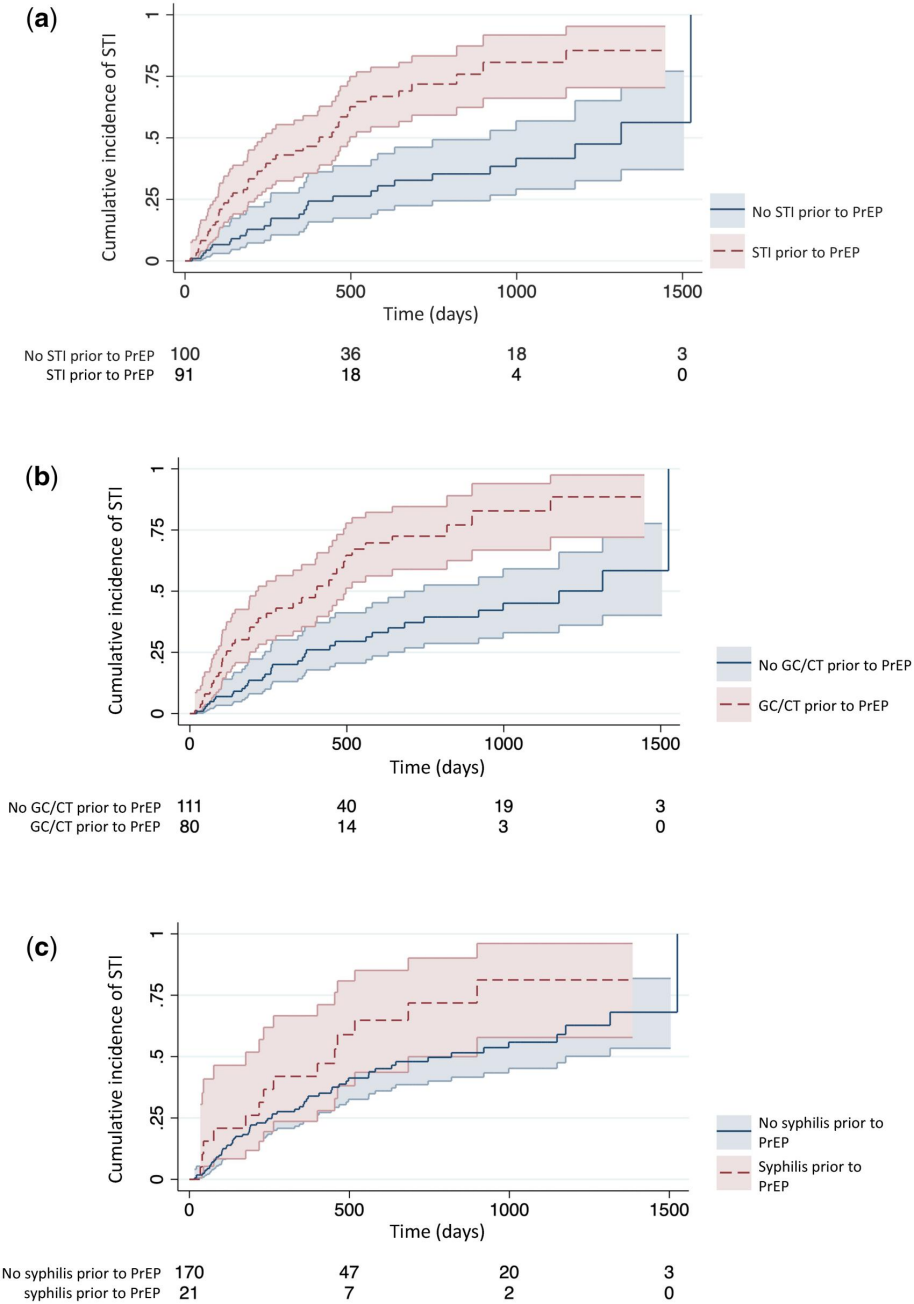
Cumulative incidence of STIs during follow-up among MSM taking PrEP (*N* = 191) stratified by (a) Any STI prior to PrEP initiation (Gonorrhoea or Chlamydia up to 2 years prior, syphilis up to 5 years prior), (b) Gonorrhoea or Chlamydia up to 2 years prior to PrEP initiation, and (c) Syphilis up to 5 years prior to PrEP initiation.

When comparing the two groups concerning bacterial STI prior to PrEP initiation, the median time until the first bacterial STI during follow-up is more than twice as high in the group who did not have an STI diagnosis prior to PrEP initiation (Fig. 1a). The effect was weaker but evident also when examining chlamydia and gonorrhoea separate from syphilis prior to PrEP (Fig. 1b). This translates into a crude hazard ratio of 2.91 (CI 95% 1.81–4.69) for any STI diagnosis prior to PrEP, 2.81 (CI 95% 1.77–4.45) for chlamydia or gonorrhoea diagnosis 2 years prior to PrEP, and 1.67 (CI 95% 0.93–2.99) for Syphilis diagnosis 5 years prior to PrEP initiation (Table 3). Due to likely interaction between poppers use and group sex, we chose to only include group sex in the final model. In this model, when adjusting for group sex, age, educational level and number of partners, the hazard ratio for having had a bacterial STI prior to PrEP initiation was 2.87 (CI 95% 1.72–4.80). In the fully adjusted model, the included covariate group sex remained significant with the hazard ratio (2.06, CI 95% 1.03–4.13). Possible interaction between included covariates were examined, but none could be confirmed.

**Table 3.** Crude and adjusted hazard ratio (HR) of STI diagnosis during follow-up among MSM taking PrEP (*N* = 191)

	Crude HR	CI (95%)	*P*	Adjusted HR	CI (95%)	*P*
Combined: STI diagnosis prior to PrEP	2.91	1.81–4.69	<0.001	2.87	1.72–4.80	<0.001
GC/CT diagnosis 2 years prior	2.81	1.77–4.45	<0.001	–		
Syphilis diagnosis 5 years prior	1.67	0.93–2.99	0.084	–		
Above 35 years of age	1.01	0.63–1.62	0.981	0.88	0.53–1.47	0.634
University education	1.04	0.63–1.71	0.888	0.85	0.51–1.43	0.549
18 male partners or more, 12 months	1.55	0.95–2.53	0.077	1.07	0.60–1.91	0.826
Group sex, 12 months	2.12	1.18–3.81	0.011	2.06	1.03–4.13	0.041
Used poppers during sex, 12 months	1.97	1.15–3.40	0.014			
Used non-injected hard drugs during sex, 12 months	0.88	0.44–1.77	0.720			
Used erectile dysfunction drugs during sex, 12 months	1.44	0.89–2.31	0.138			
Sex abroad, 12 months	1.29	0.76–2.20	0.350			

## Discussion

The current study shows increased incidences of bacterial STIs during PrEP. Having had a STI prior to PrEP (2-year prior for gonorrhoea and chlamydia, 5 years prior for syphilis) and experience of group sex in the past 12 months were associated with increased risk of STI acquisition after PrEP initiation. Our data suggest that the MSM who used PrEP in Sweden during the initial years when PrEP became available (2018–2023) are MSM with increased STI risk and thus are highly relevant from a bacterial STI preventive perspective. There are however a number of factors that needs consideration. When evaluating STI trends in relation to PrEP use, it is important to consider the pre-existing trends [18], and while this study noted an increase in STI incidences, there was an overall increase in bacterial STIs in Sweden during the follow-up time. The number of syphilis cases among MSM increased by 45% between 2017 and 2021, and overall the incidence of syphilis has increased by an average of 15% annually since 2010 [19]. The same trend of an average annual 15% increase in incidence was observed for gonorrhoea in the period prior to Covid-19 and in the region of Skåne (including the Malmö study site), the number of new cases of gonorrhoea reported increased by 74% between 2021 and 2022 [19]. Moreover, it is likely that participants seek PrEP in periods of increased risk. Indeed, testing frequencies increased by 75% for gonorrhoea/chlamydia, indicating that increased incidences at least partly might be explained by higher testing frequencies.

Overall STI incidence during follow-up in this study was around 81 infections per 100 person-years, which is comparable to previous studies from France, Canada, and the Netherlands [20, 21]. Traeger *et al.* [22] note that their meta-analysis findings suggest that STIs increase after PrEP initiation. However, in their longitudinal study from 2019, they also note that STIs were concentrated among a subset of PrEP users, which is further supported by the findings in the current study [23]. While indeed the episodes of infections increased after PrEP initiations in the current study, the number of individuals getting diagnosed with gonorrhoea or chlamydia actually decreased slightly from 42 individuals per year prior to PrEP to 39 per year during follow-up. We also note that episodes of new infections commonly occur, with as many as 18 infections diagnosed in one participant during follow-up. This points to the importance of identifying those at higher risk of infection, to ensure timely screening of bacterial STIs during PrEP use.

The current study set out to examine if STI diagnosis prior to PrEP initiation could be a useful predictor to identify PrEP users at increased risk of bacterial STIs. We could establish that with a prior STI diagnosis, the risk of a new episode of STI after PrEP initiation was almost 3-fold compared to those not having had previous STI diagnosis, suggesting that it is a predictor of clinical relevance. Interestingly, when examining behavioural variables, having had group sex in the preceding 12 months, doubled the risk of bacterial STI during follow-up. This stresses the importance of combining behavioural and clinical assessments when planning PrEP-related care and follow-up. A similar risk increase was reported in Australia where self-reported STI in the 3 months preceding PrEP enrolment increased the risk of STI in a study population with similar demographics to our study [23].

According to Swedish and many international guidelines, PrEP users are screened every 3 months for STIs (3 × 3). If priorities have to be made regarding follow-up frequency, a logical strategy would be to concentrate on the individuals with a higher likelihood of contracting bacterial STIs and reduce the testing frequency for those with fewer risk factors. However, when studied in a PrEP cohort in Amsterdam, it was concluded that reducing screening frequency would likely result in delayed STI diagnoses [24] indicating that caution has to be taken if adopting such an approach. In addition, regular STI screening visits offer an opportunity to offer other health interventions including counselling, addressing harmful alcohol use, drug use, and offering referrals as part of comprehensive PrEP programmes. A recent randomized controlled trial (RCT) comparing the effect of screening (3 × 3) versus non-screening for gonorrhoea and chlamydia on the incidence of these infections in MSM and transgender women taking PrEP in Belgium failed to show that non-screening is non-inferior to 3 × 3 screening. The RCT reports a higher incidence of chlamydia in the non-screening arm, no difference in gonorrhoea infections, and lower antibiotic use in the non-screening arm. Data that calls for further evaluation of screening frequency in PrEP programmes [25, 26].

While STIs are a concern, PrEP also gives a sense of self-control, increases freedom to explore, and facilitates sexual exploration and pleasure [10]. It has been argued that too much attention is directed towards (medical) risk behaviours and changes in behaviours in relation to PrEP use. It would also include our current study. A focus on these behavioural factors may risk obfuscation of other factors such as the opportunities for freedom, and their importance in decision-making among PrEP users which needs to be further investigated [27]. Nevertheless, the overall trend of increasing incidences of bacterial STIs in Sweden and elsewhere is a valid public health concern that needs to be addressed.

### Methodological considerations

The number of study participants limited statistical power and suitable analyses which needs to be taken into account when interpreting the results. While the interaction between covariates in the Cox proportional hazard regression models could not be confirmed, it cannot be excluded due to the limited sample size. The test rate for syphilis could not be calculated as tests may derive from control tests after active infections. Journal review could not distinguish between screening or control as cause for performed tests.

The study uses self-reported questionnaire data on sensitive topics such as sexual behaviours and drug use which might have an increased risk of introducing social desirability bias during data collection. To minimize this, participants were given the questionnaire in a private environment at gay-friendly venues with extensive experience of sexual health and efforts of ensuring privacy when answering the questionnaire [28]. Further recall bias may occur, it may be difficult for some to recall and estimate the number of sexual partners and condom use in the last 12 months. To help address this, participants were encouraged to take their time to estimate this, use their mobile phones as aid, and evaluate the number of partners per calendar month to facilitate a correct estimate. Nevertheless, it is not unlikely that some behaviours are under-reported, such as drug use.

This study took part during the Covid-19 pandemic and while testing services never closed for individuals already on active PrEP use, we cannot control for lesser variations in the availability of testing or for changes in sexual contact patterns during the epidemic period. As the study was performed in three large cities in Sweden, there might be a lack of representativeness of PrEP users from other locations in Sweden where PrEP is prescribed.

## Conclusion

The current study found that STI prior to PrEP initiation and group sex were associated with STI acquisition after initiation of PrEP, factors that could be considered if needing prioritizing the frequency of STI screening.

## Data Availability

Data are available upon reasonable request. The full anonymized dataset will be shared upon reasonable request to SS in order to protect participant confidentiality. This is motivated by that the dataset contains sensitive information on a rather small sample of a stigmatized population (MSM), and sociodemographic data might theoretically make the data traceable.
